# Ameliorative Effects of Gut Microbial Metabolite Urolithin A on Pancreatic Diseases

**DOI:** 10.3390/nu14122549

**Published:** 2022-06-20

**Authors:** Kailin Li, Yao Xiao, Ji Bian, Lin Han, Caian He, Emad El-Omar, Lan Gong, Min Wang

**Affiliations:** 1 College of Food Science and Engineering, Northwest A&F University, Xianyang 712100, China; ailklin@nwsuaf.edu.cn (K.L.); changyangxiaoyao@163.com (Y.X.); hanlin730@163.com (L.H.); caian.he@nwafu.edu.cn (C.H.); 2 Kolling Institute, Sydney Medical School, Royal North Shore Hospital, University of Sydney, St. Leonards, NSW 2065, Australia; jbia3972@uni.sydney.edu.au; 3 Microbiome Research Centre, St George and Sutherland Clinical School, University of New South Wales, Sydney, NSW 2052, Australia; e.el-omar@unsw.edu.au

**Keywords:** urolithin A, pancreas, autophagy, microbiota, diet

## Abstract

Urolithin A (Uro A) is a dietary metabolite of the intestinal microbiota following the ingestion of plant-based food ingredients ellagitannins and ellagic acid in mammals. Accumulating studies have reported its multiple potential health benefits in a broad range of diseases, including cardiovascular disease, cancer, cognitive impairment, and diabetes. In particular, Uro A is safe via direct oral administration and is non-genotoxic. The pancreas plays a central role in regulating energy consumption and metabolism by secreting digestive enzymes and hormones. Numerous pathophysiological factors, such as inflammation, deficits of mitophagy, and endoplasmic reticulum stress, can negatively affect the pancreas, leading to pancreatic diseases, including pancreatitis, pancreatic cancer, and diabetes mellitus. Recent studies showed that Uro A activates autophagy and inhibits endoplasmic reticulum stress in the pancreas, thus decreasing oxidative stress, inflammation, and apoptosis. In this review, we summarize the knowledge of Uro A metabolism and biological activity in the gut, as well as the pathological features and mechanisms of common pancreatic diseases. Importantly, we focus on the potential activities of Uro A and the underlying mechanisms in ameliorating various pancreatic diseases via inhibiting inflammatory signaling pathways, activating autophagy, maintaining the mitochondrial function, and improving the immune microenvironment. It might present a novel nutritional strategy for the intervention and prevention of pancreatic diseases.

## 1. Introduction

Urolithin A (3,8-dihydroxy-dibenzoprranone, Uro A) and its glucuronic acid derivatives are the main urolithins in the blood and urine of mammals following the consumption of ellagitannins (ETs) and ellagic acid (EA). Urolithins are 6H-dibenzopyran-6-one derivatives (or aglycons) that were first isolated from natural sources (scent glands of beaver) in 1949 [1]. In vitro and in vivo experiments have shown that Uro A, as a natural active substance, has various health benefits, including antioxidant, anti-inflammatory, and cancer inhibition. Interestingly, these activities of Uro A are higher than those of Uro A precursor compounds (ETs and EA) for both disease prevention and treatment. Therefore, Uro A has broad prospects in clinical applications. For example, the benefits of Uro A supplementation in muscles have been supported by recent clinical trials in older adults [2,3]. Additionally, Uro A improves muscle strength, exercise performance, and biomarkers of mitochondrial health in middle-aged adults [4].

Additionally, studies have demonstrated the safety of direct oral Uro A. The genotoxicity tests also show that Uro A is not genotoxic. Consistent with the results obtained by the population experiment, there was no significant systemic and tissue toxicity in rats after the continuous intragastric (i.g.) administration of Uro A for 90 days [5]. This study also revealed that the No Observed Adverse Effect Level (NOAEL) was determined to be 3451 mg/kg bw/day in male rats and 3826 mg/kg bw/day in female rats, which would be equivalent to a human-equivalent dose of approximately 557 mg/kg bw/day in males and 617 mg/kg bw/day in females [5]. Because of its effectiveness and safety, Uro A has been recognized as safe by the US Food and Drug Administration and was approved for use as a food ingredient.

The pancreas is the center that regulates energy consumption and metabolism through secreting digestive enzymes and hormones [6]. The exocrine and endocrine glands form the morphologic and functional parts of the pancreas. A total of 95% of the exocrine pancreas consists of acinar cells and duct cells [7]. Endocrine cells are spherically clustered into the islet of Langerhans to form the endocrine pancreas [8]. Many pathophysiological conditions can hamper endocrine and exocrine pancreas functions, such as pancreatitis, pancreatic cancers, and diabetes mellitus (DM). In this review, we summarize the knowledge of Uro A metabolism and biological activity. Moreover, we discuss the pathological features and mechanisms of common pancreatic diseases. Finally, Uro A’s potential effect and mechanisms on the pancreas are further explored.

## 2. Urolithin A

### 2.1. Intestinal Microbial Metabolite Urolithin A

**Figure 1:**
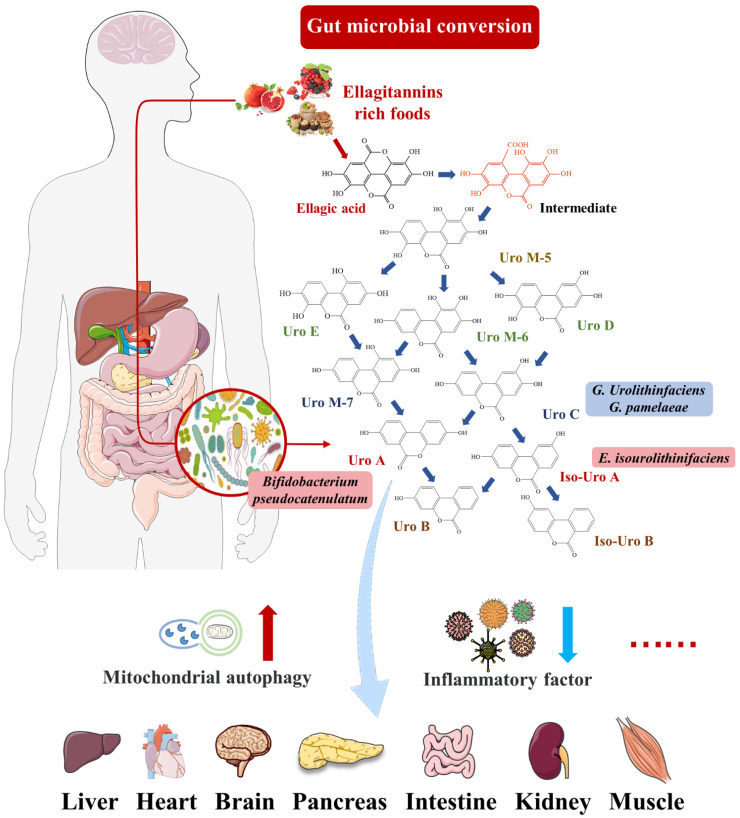
Uro A can travel through the bloodstream to peripheral tissues, including pancreas, brain, liver, and muscle tissue, and perform biological activities, such as preventing inflammation and increasing mitophagy.

EA is a natural polyphenol that mainly exists in its condensed form—ETs. They are found in various berries, nuts, and seeds and are consumed in large quantities by mammals, including humans [9]. There is a correlation between ETs and health effects. However, EA is difficult to be absorbed directly by the gastrointestinal tract because it is a highly polar macromolecule. Thus, ET levels in tissues and plasma are insufficient to achieve the concentration required for its biological activity [10]. As shown in Figure 1, it has been shown that most ETs and EA react with the abundant gut microbiota to produce new metabolites with powerful health functions, such as urolithins. Urolithins are dibenzopyran-6-one derivatives with different hydroxyl substitutions, which can be considered a combination of coumarin and isocoumarin [11]. Natural urolithins were first isolated from the scent glands of beavers in 1949 and were gradually proved to be widespread in microbial, plant, animal, and human feces [1]. In terms of chemical structure, urolithins are produced from EA by lactone ring-opening and decarboxylation, and by gradually removing hydroxide radicals from various sites via dehydroxylase [12]. Common urolithins include Uro A, Uro B, iso-Uro A, iso-Uro B, Uro C, and Uro D. Uro A has attracted attention due to its mitochondrial autophagy activation, anti-aging and antioxidant properties, and other functions.

There were significant individual differences in the production and excretion of Urolithins [10]. Urolithin metabotypes (UMs) can be divided into three phenotypes (UM-A, UM-B, and UM-0) based on the different abilities of human subjects to excrete urolithins. UM-A produced Uro A; UM-B mainly produced iso-Uro A and Uro-B; and UM-0 did not produce Uro-A, iso-Uro A, or Uro B [13]. The distribution of UM-A and UM-B was affected by aging, with UM-A decreasing from 85% to 55% after adulthood [14]. Specific intestinal microflora can convert ETs and EA consumed by some people into urolithins [15]. Selma et al. reported that the abundance of *Gordonibacter* in the feces of UM-A individuals was higher than those of UM-B and UM-0 individuals [16]. In addition, two strains from the genus *Gordonibacter* with a Uro-C-producing ability were identified: *G. urolithinfaciens* and *G. pamelaeae* [17]. *E. isourolithinifaciens* was a strain from the genus *Ellagibacter* with the ability to produce iso-Uro A [18]. Additionally, Uro A could be produced by *Bifidobacterium pseudocatenulatum* from the genus *Bifidobacterium* [19]. The other studies consistently showed that Uro A and iso-Uro A could be found in fermentation broth inoculated with the fecal flora of two volunteers in vitro [12,20]. The *Clostridium leptum* group from the *Firmicutes* phylum was identified as the dominant flora in the fermentation broth, followed by *Bacteroids* and *Prevotella* [20]. These bacteria with the ability to produce urolithins can be used as novel probiotics in functional food and health products.

To date, all the strains used to synthesize Uro A by fermentation in vitro are mixed strains without success with single strains. This may be because multiple bacteria are involved in the formation of intermediates during EA metabolism. Uro A prepared by biological fermentation is safe and has a wide range of raw materials. However, its purity is lower than chemical synthesis.

### 2.2. Pharmacokinetics of Urolithin A

The data suggested that, after the direct oral administration of Uro A (250–1000 mg) in healthy, older adults, its maximum plasma concentration (C _max_) and area under the curve (AUC) were dose-dependent. Furthermore, after oral administration, the highest C _max_ was Uro A-glucuronide (1500–3000 nM), followed by Uro A-sulfate (200–400 nM) and Uro A (4–7 nM). All three substances exhibited similar kinetics, with peak concentrations in plasma at 6 h (T _max_) after dosing. The half-life (t _1/2_) of Uro A and Uro A-glucuronide ranged from 17 to 22 h, while the t _1/2_ of Uro A-sulfate was relatively long at 25–88 h. They were completely eliminated from plasma circulation within 72 to 96 h [2]. Animals were also treated by either i.g. or intravenous (i.v.) administration of ^14^C radiolabeled Uro A. After i.g. administration of Uro A, the majority of them were excreted via the feces. Total Uro A reached C _max_ at around 3 h and then again around 6 or 7 h. In contrast, i.v. administration resulted in the urine as the main excretion route. Moreover, the results show that absorption after i.v. administration is higher than that after i.g. administration [5]. In conclusion, these pharmacokinetic data indicated that Uro A had favorable bioavailable profiles. In particular, after oral administration of EA (50 mg/kg) in rats, the plasma levels peaked at about 0.5 h, with a C _max_ value of 93.6 ng/mL, showing that EA was poorly absorbed after oral administration. The pharmacokinetic profile of EA fitted to a two-compartment model with t _1/2 α_ 0.25 h and t _1/2 β_ 6.86 h, respectively [21].

Uro A-glucuronide is the main existing form of Uro A in blood circulation. Uro A-glucuronide is a macromolecule with many polar groups, making its transmembrane transport ability extremely low. However, a recent study showed the deconjugation of Uro-A glucuronide to Uro-A in the Sprague Dawley rat model of systemic inflammation induced by lipopolysaccharide (LPS). Therefore, the ratio of Uro A-glucuronide to Uro-A in the intestine, liver, kidney, bladder, spleen, lung, muscle, and urine was significantly decreased [22]. For instance, 8 h after a single oral administration of 2000 mg of Uro A, its parent state was mainly detected in skeletal muscle tissue (6 ng/g) [2].

### 2.3. Biological Activity of Urolithin A

Due to the strong biological activity of Uro A, studies on it have considerably increased in recent years. Many in vivo and in vitro experiments have shown that Uro A can improve oxidative stress, cognitive impairment, are anti-inflammatory and anti-aging, activate mitochondrial autophagy, and enhance intestinal barrier and other functions.

#### 2.3.1. Anti-Inflammatory and Improved Oxidative Stress

Uro A can improve neuroinflammation, renal toxicity, and more. DaSilva et al. found that Uro A significantly improved LPS-induced BV2 cell inflammation [23]. Uro A reduces nitric oxide (NO) levels and down-regulates the expression of inflammatory factors, including tumor necrosis factor (TNF)-α, interleukin (IL)-6, IL-1β, inducible nitric oxide synthase (iNOS), and cyclooxygenase-2 (COX-2). This effect was achieved through the inhibition of nuclear factor kappa-B (NF-κB), mitogen-activated protein kinase (MAPK), and activation of the Akt signaling pathway [23]. At the same time, Uro A reduced the expression of reactive oxygen species (ROS) in neuro-2a Cells induced by H_2_O_2_ by enhancing the activity of antioxidant enzymes [24]. Additionally, the study showed that Uro A significantly ameliorates cisplatin-induced nephrotoxicity in mice via modulating inflammation and oxidative stress [25]. To be specific, Uro A significantly reduced neutrophilic gelatinase-associated lipoprotein (NGAL), blood urea nitrogen (BUN), creatinine, and urinary kidney injury molecule-1 (KIM-1) in serum.

#### 2.3.2. Anti-Aging

As a natural active substance, Uro A shows great anti-aging potential. Ryu et al. first demonstrated that Uro A improved mitochondrial function by inducing mitochondrial autophagy in C. elegans, thereby prolonging its lifespan and maintaining the normal activities of nematodes during senescence [26]. Furthermore, Uro A significantly inhibited the impaired autophagy in aging mice caused by D-galactose-induced overexpression of miR-34a [27]. They proved that Uro A upregulates the Sirt1 signaling pathway and down-regulates the mTOR signaling pathway to activate autophagy. In human skin fibroblasts, Uro A can reduce the expression of matrix metalloproteinase-1(MMP-1) and increase the expression of type-I collagen in senescent cells [28]. Uro A reduced ROS in senescent cells by activating nuclear factor erythroid 2-related factor 2 (Nrf2)-mediated antioxidant system. Importantly, Uro A safeguarded against physiological decline, as illustrated by improving muscle function in young animals, demonstrating the benefits of Uro A in healthy environments [26,29]. However, whether Uro A can delay pancreatic aging or enhance pancreatic function in healthy people and its mechanism remains unclear.

#### 2.3.3. Regulation of Metabolic Homeostasis

The incidence of metabolic diseases, such as insulin resistance (IR), obesity, metabolic syndrome, and DM increases. As a metabolite of foodborne EA via gut microbiota, Uro A was thought to have the activity of improving glucose and lipid metabolism disorders. In a high-fat-diet (HFD)-induced IR model, Uro A significantly reduced fasting blood glucose, serum triglycerides, free fatty acids, and increased adiponectin content [30]. Uro A interfered with cholesterol metabolism by regulating the expression of miR-33a and ERK/AMPKα/SREBP1 signaling pathways [31]. Xia et al. indicated that Uro A inhibited obesity induced by HFD via enhancing thermogenesis in brown adipose tissue and promoting the browning of white adipose tissue [32]. Overall, Uro A played an important role in weight control, glucose homeostasis, and lipid metabolism balance.

#### 2.3.4. Improve Alzheimer’s Disease and Cognitive Impairment

Alzheimer’s disease (AD) has no good treatment in the world. Many pathogenic factors of AD exist, such as gene mutation, an unhealthy lifestyle, and brain trauma. Uro A is active against cognitive impairment and AD. Gong et al. proved that Uro A ameliorated cognitive impairment, attenuated neuronal apoptosis, promoted neurogenesis, and decreased accumulation of microglia and astrocytes in the APP/PS1 mouse AD model [33]. Impaired mitochondrial autophagy is one of the mechanisms resulting in AD. As an activator of mitochondrial autophagy, Uro A can inhibit Tau hyperphosphorylation through PINK-1, PDR-1, and DCT-1 signaling pathways, thereby restoring memory impairment in the AD model in C. elegans [34]. Long-term metabolic disorders also contribute to the development of AD. Lee et al. found that Uro A significantly reduced mitochondrial calcium overload and the accumulation of mt-ROS induced by high glucose and inhibited amyloid β-protein (Aβ)-related enzymes [35].

Studies have shown that Uro A also has other activities, such as protecting the intestinal barrier to maintain intestinal integrity, inducing cancer cell death, and improving cardiac dysfunction. It is well known that the function of gut microorganisms and gut barriers to human health are indisputable. Uro A exerted its barrier functions by activating aryl hydrocarbon receptor (AhR)-Nrf2-dependent pathways to upregulate epithelial tight junction proteins, which could reduce colon inflammation [36]. Moreover, the activities of Uro A in improving cerebral ischemia-reperfusion injury and Parkinson’s disease have also been reported. Uro A has solid biological activity both in vivo and in vitro.

In most cases, Uro A played a role as an activator of mitochondrial autophagy. Uro A also played a role in improving endoplasmic reticulum (ER) stress and regulating gene expression. More possible biological activities and their specific mechanisms need to be further explored.

## 3. Pathogenic Mechanisms of Pancreatic Diseases

The pancreas is a primary target of free radicals due to its high synthetic and secretory activities, which result in oxidative damage [37]. In addition, pathophysiological changes, such as self-digestion, inflammatory response, intracellular Ca^2+^ overload, ER stress, mitochondrial dysfunction, and weakening of the immune system, can also impair endocrine and exocrine pancreas functions. The most common life-threatening pancreatic diseases include pancreatitis, DM, and pancreatic cancer.

### 3.1. Pancreatitis

Acute pancreatitis (AP), usually accompanied by acinar cell necrosis, is one of the most common diseases among gastroenterology disorders [38,39]. AP refers to intrapancreatic trypsinogen activation caused by gallstones, hypertriglyceridemia, metabolic abnormalities, obesity, and alcoholic intemperance, which is characterized by early local inflammatory damage of the pancreas and may progress to the serious systemic inflammatory response [40,41]. The global incidence in AP is 33.74 cases (95% confidence interval (CI): 23.33–48.81) per 100,000 individuals per year, and it is rising continuously at about 3.4% a year [42,43]. The incidence is not statistically significant between the sex, but increases with age [44]. Patients with mild AP have a good prognosis, but moderately severe AP or severe AP patients account for 15–20%, resulting in mortality rates as high as 30% because of persistent organ failure and pancreatic necrosis [45]. The pathogenesis of AP is associated with complex intra-acinar events, such as autophagy, oxidative stress, mitochondrial dysfunction, and ER stress [46]. Furthermore, inflammatory responses, including the recruitment of immune cells, activation of damage-related molecular patterns, and release of various inflammatory cytokines and chemokines, have been partly involved in the development of AP [47]. Based on recent studies on the effects of natural active substances on AP, three major signaling pathways, including NF-κB, Nrf2, and MAPK, have received great interest and attention from researchers and industries [47]. (1) During the development of AP, NF-κB is rapidly activated in pancreatic acinar cells, followed by significant increases in inflammatory cytokines and chemokines, which can affect vascular permeability and lead to thrombosis, bleeding, and tissue necrosis [48,49]. (2) Oxidative damage and inflammatory cascade amplification are essential factors that cause AP to evolve into severe AP [50]. Nrf-2 is a key factor of the endogenous antioxidant pathway, which is involved in a series of physiological activities of inflammatory response. Nrf-2 upregulates the expression of heme oxygenase 1(HO-1), quinone oxidoreductase 1 (NQO1), superoxide dismutase (SOD), and other antioxidant enzymes through the Nrf2/ antioxidant response element (ARE) signaling pathway [51]. These antioxidant proteins exert cytoprotective effects against pancreatic acinar cell injuries. It also down-regulates the expression and secretion of malonic dialdehyde (MDA), myeloperoxidase (MPO), C-reaction protein (CRP), and other inflammatory proteins. (3) Three predominant members of the MAPK family, c-Jun NH2-terminal kinase (JNK), extracellular signal-regulated kinase (ERK), and p38 MAPK, are upregulated to mitigate early AP progression by reducing pancreatic acinar cells damage and inhibiting inflammation [47].

Chronic pancreatitis (CP) is a complex disease characterized by a persistent or repeated inflammation of the pancreas that leads to progressive and irreversible morphologic changes causing impairment of pancreatic function [52]. Functional consequences include DM (endocrine insufficiency) and dyspepsia (exocrine insufficiency). CP develops in 36% (95% Cl: 20–53%) of patients with recurrent AP [40]. Similar to AP, CP mainly affects middle-aged and elder patients [44]. The crude mortality of CP is 0.09 (95% Cl: 0.02–0.47) per 100,000 individuals per year [42]. Additionally, the majority of patients with CP die from non-pancreatitis causes, such as cancer and cardiovascular disease [53]. Early CP is difficult to diagnose because the lesions are subtle and similar to other diseases [54]. The typical manifestations of later CP are focal necrosis, fibrosis, irregular enlargement of the pancreatic duct, pseudocysts, intraductal calculi, and calcifications [55]. CP’s potential causes include toxic factors (alcohol or smoking), metabolic abnormalities, genetics, impaired autoimmune, and disease [56]. The pathological changes of CP are quite complex, including acinar cell damage, acinar stress response, ductal dysfunction, and persistent or altered inflammation, but these mechanisms are not fully understood [57].

### 3.2. Pancreatic Cancer

Pancreatic cancer is a highly fatal disease with a 5-year survival rate of about 10% in the United States [58]. The most common and deadliest form of pancreatic cancer is referred to as pancreatic ductal adenocarcinoma (PDAC) [59]. Although the incidence of PDAC has been increasing year by year, the mortality rate has not decreased significantly due to late diagnosis, early metastasis, and limited response to chemotherapy or radiotherapy [60]. A major hallmark of PDAC is the presence of several activated oncogenic signaling pathways that contribute to the aggressiveness of disease and therapeutic resistance. For example, more than 90% of PDAC patients have K-RAS mutations that activate downstream pathways, such as phosphatidylinositol-3-kinase (PI3K)-Akt, to promote tumor genesis [61]. This pathway has been demonstrated in mice as well. Furthermore, in the pancreas of mice expressing PI3KCA mutations, acinar-to-ductal metaplasia (ADM) and pancreatic intraepithelial neoplasms (PanINs) progress to invasive PDAC [62]. PI3K-activated phosphorylation of Akt (the serine-threonine kinase of the AGC kinase family) also affects the expression of anti-apoptotic and cell-cycling-related proteins and transcription factors. Approximately 60% of PDAC patients experience increased Akt activity due to hyperphosphorylation, while Akt overexpression due to gene amplification is recorded in 10–20% of PDAC patients [63]. In addition, the complex PI3K signaling network plays a role in activating mTOR, NF-κB, GSK3β, p27, and Bad-Bax pathways [64]. 

Immune evasion is also a major obstacle to PDAC treatment. Common evasion mechanisms include impaired antigen presentation due to mutations or loss of heterozygosity of the major histocompatibility complex class I (MHC-I) [65]. In PDAC, MHC-I is selectively degraded by lysosomes through an autophagy-dependent mechanism. PDAC can utilize its high basal autophagy levels to support its metabolism and maintain tumor growth. Notably, the inhibition of autophagy can restore MHC-I surface levels, improve antigen presentation, enhance anti-tumor T-cell responses, and reduce tumor growth [66]. What is more, the inhibition of autophagy also results in the loss of SLC7A11 on the plasma membrane and increases its localization at the lysosome in a mTORC2-dependent manner [67].

Metastatic PDAC has a lower infiltration of total T cells than resectable primary PDAC, proving that metastatic PDAC has poor immunogenicity. Furthermore, the number of CD68 (+) macrophages and VISTA (+) cells is significantly increased in the pancreatic stromal region of metastatic PDAC patients. Hence, VISTA may be a relevant immunotherapy target for the effective treatment of PDAC patients [68]. Ordinary care drugs, such as FOLFIRINOX and gemcitabine plus nab-paclitaxel, have limited clinical effects and are poorly tolerated by patients due to toxic side effects [69]. Therefore, there is an urgent need to develop new therapies to reduce the PDAC burden without significant off-target effects.

### 3.3. Diabetes Mellitus

Diabetes mellitus (DM) is a chronic metabolic disease characterized by elevated blood sugar levels over time leading to damage to the heart, vasculature, eyes, kidneys, and nerves [70]. According to the International Diabetes Federation, the global number of DM patients has increased in recent decades and is predicted to ascend to 642 million by 2040 [71]. Type 2 diabetes mellitus (T2DM) is the most common type of DM, accounting for about 90% of all cases [72]. T2DM is characterized by insufficient insulin secretion by β cells, IR, and inadequate compensatory insulin secretion [73]. Early on, when cells become IR, β cells secrete high amounts of insulin, leading to hyperinsulinemia. In later stages, with a gradual decline in β-cell function, insulin levels are insufficient to meet increased insulin requirements, which produce hyperglycemia [74]. The link between islet cell injuries and DM has been established for several years. Restricted islet cell proliferation and regeneration as well as reduced secretion capacity lead to a decline in the accurate management of glucose homeostasis. For example, many GWAS studies attested that SNPs adjacent to the *CDKN2a/b* gene is associated with T2DM [75]. This discovery suggests that β-cell-proliferation-related genetic defects might increase the susceptibility of T2DM. 

Many studies have shown the role of autophagy in β-cell function and survival. Autophagy can participate in the catabolic process of removing cytotoxic proteins and damaged organelles in cells under stress conditions to promote the survival of β cells under conditions conducive to cell death, including nutrient depletion, inflammation, hypoxia, and mitochondrial damage [76]. For example, Quan et al. showed that β-cell-specific autophagy-related 7 (Atg7)-null mice showed hypo-insulinemia and hyperglycemia, which induced DM [77]. In addition, the macrophage populations shift their polarity to a more inflammatory phenotype during islet inflammation, increasing the amplification of islet inflammation [78]; for instance, in many macrophage marker CD68+ cells near islets from DM patients, which is associated with decreased insulin immunoreactivity and increased amyloid deposits [79,80,81]. Hence, inflammation and immune damage play essential roles in the pathogenesis of DM and contribute to β-cell dysfunction in diabetes mouse models. In recent years, gut microbiota dysbiosis has been widely discussed as a driver of diabetes pathophysiology. Metformin, one of the most commonly used hypoglycemic agents, is known to alter the composition of gut bacteria [82]. In conclusion, DM, as a systemic metabolic disease, has complex pathophysiological driving factors. Therefore, we are supposed to fully consider the effect of islet cell injuries on DM.

## 4. Effects of Urolithin A on Pancreatic Diseases

### 4.1. Reduces the Expression of Pancreatic Inflammatory Factors

The inflammatory microenvironment of the pancreas led to pancreatitis and was the main reason for the decline in endocrine function [83]. Some researchers suggested that if β cells would express high levels of NF-κB signaling marking, cells’ proliferative and regenerative potential were reduced. The NF-κB-expressed β cells also emerged with a premature upregulation of *socs2*, a gene that inhibits proliferation [84]. It has been widely reported that EA can inhibit pancreatic inflammation (Table 1). In an experimental model of spontaneous chronic pancreatitis, male Wistar Bonn/Kobori rats were fed a diet supplemented with EA (100 mg/kg body weight/day) for ten weeks. They found that EA attenuated pancreatic inflammation and fibrosis by increasing pancreatic weight and decreasing MPO activity (a neutrophil infiltration index), collagen content, transforming growth factor-β1 (TGF-β1) expression, activated pancreatic stellate cells (PSCs), and ED-1-positive cells [85]. Masamune et al. also reported that EA inhibited the production of monocyte chemoattractant protein-1 (MCP-1) and activation of activator protein-1 (AP-1) and MAPK in PSCs, all induced by interleukin (IL)-1β and TNF-α [86]. Meanwhile, EA inhibited PDGF-BB-induced tyrosine phosphorylation of PDGF P-receptors and the downstream ERK and Akt activation in PSCs. In particular, EA inhibited ROS production in PSCs in response to TGF-β1 or platelet-derived growth factor (PDGF) [87].

**Figure 2:**
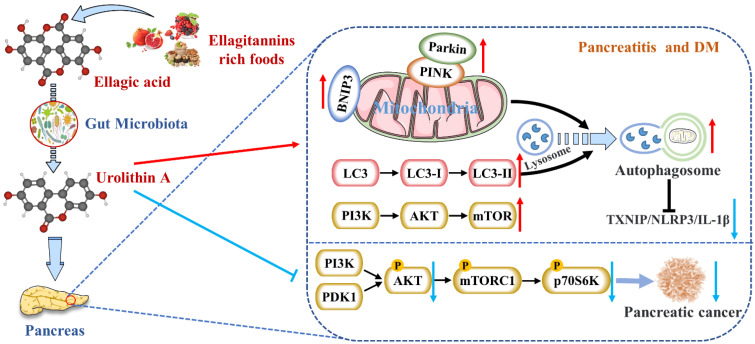
Uro A is metabolized by gut microbiota after ingestion of ETs and EA in mammals and has multiple potential health benefits. Uro A can attenuate pancreatic diseases by inhibiting inflammatory signaling pathways, activating autophagy, maintaining the mitochondrial function, and improving the immune microenvironment.

Although EA had many promising developments, it was poorly absorbed in the human gut, limiting its anti-inflammatory effects. As mentioned above, EA was metabolized by microorganisms into a series of downstream compounds, such as Uro A [5]. A well-known effect of preclinical models exposed to Uro A was the attenuation of harmful inflammatory responses [94]. Uro A showed more potent anti-inflammatory properties than EA or ETs, suggesting that it might be the main compound for treating AP or CP (Table 1). The anti-inflammatory effects were first reported to reduce the mRNA and protein levels of inflammatory marker COX-2 in rats with acute colitis [95]. Zhang et al. had firstly reported that Uro A inhibited the thioredoxin-interacting protein (TXNIP)/Nod-like receptor family pyrin domain containing 3 (NLRP3)/IL-1β inflammation signal in MIN6 β cells by modulating AMPK (Figure 2) [88]. Finally, they testified that Uro A also down-regulated the protein kinase RNA (PKR)-like ER kinase (PERK) and promoted AMPK phosphorylation [96]. The latest research showed that Uro A can attenuate the severity of alcohol-associated chronic pancreatitis (ACP) in C56BL6/J mice by regulating the PI3K/AKT/mTOR signaling axis [89].

Nevertheless, studies on reducing pancreatic inflammation by Uro A had only been verified in animals and cells without clinical studies. The upstream mediators of Uro A’s anti-inflammatory effects, including the NF-κB and AhR-Nrf2 pathways, were mainly studied in vitro [36]. Nevertheless, the mechanisms of Uro A action in the context of inflammation seemed to vary with tissues and conditions. Hence, the differences in Uro A’s mitigation degree and mechanism on AP and CP need to be further explored.

### 4.2. Activates Autophagy and Maintains Mitochondrial Function in the Pancreas

Mitochondrial damage, such as the loss of mitochondrial DNA (mt DNA) integrity, the alteration of mitochondrial morphology, and dysfunction, can lead to cellular senescence and apoptosis [97]. On the one hand, mitochondria acted as both nutrient sensors and signal generators for insulin secretion in β cells. Moreover, nutrients can inhibit the ATP-sensitive K^+^ (K_ATP_) channel and then enhance insulin secretion either by acting as substrates for mitochondrial ATP synthesis (the triggering pathway) or by regulating Ca^2+^ channels (the amplifying pathway). On the other hand, mitochondria were the primary source of reactive oxygen species (ROS) at the level of the electron transport chain so that mitochondria might be the main targets of ROS damage [98]. Additionally, many studies have revealed a causal relationship between pancreatic diseases and dysregulation of mitochondrial dynamics (including fusion and fission) [97,99,100]. Thus, mitochondrial damage gave rise to decreased pancreatic function. The most consistent effect of Uro A across species, including cells, worms, mice, and humans, was improved mitochondrial health [94]. This benefit was driven by the clearance and recycling of dysfunctional mitochondria, known as selective autophagy [101]. For example, Uro A increased the expression of mitochondrial autophagy genes *lgg-1*, *pink-1,* and *pdr-1*, encoding for LC-3B, and formation of autophagosome vesicles in C. elegans [29].

Interestingly, Pink1 knockdown in microglia eliminated Uro A-mediated reductions in TNF-α and increased IL-10, suggesting that Uro A reduces neuroinflammation by inducing mitochondrial autophagy [34]. Zhang et al. also proved that Uro A inhibited glucolipotoxicity-induced ER stress and the TXNIP/NLRP3/IL-1β inflammation signal in MIN6 β cells by modulating autophagy [88]. Remarkably, the inhibitory effects of Uro A on p62 were stronger than TXNIP-inhibitor verapamil (*p* < 0.05) [102]. Uro A promoting PINK1/Parkin-mediated mitophagy was also reported in pancreatic cells of diabetic mice [90]. Therefore, Uro A restoring the correct level of mitochondrial autophagy to maintain normal mitochondrial function is highly likely to be the mechanism of Uro A reducing pancreatic diseases (Figure 2).

### 4.3. Inhibits Endoplasmic Reticulum Stress in the Pancreas

The misfolding and inhibition of protein folding in the endoplasmic reticulum (ER) lead to the aggregation of unfolded proteins, resulting in ER stress [103]. Li et al. showed that the ER stress and unfolded protein response (UPR) accompanied by the accumulation of protein aggregates emerged as a significant pathway affected by aging, specifically in β cells. Simultaneously, the transcriptomic dysregulation of UPR components was linked to activating transcription factor 6 (ATF6) and inositol-requiring enzyme 1 (IRE1) signaling pathways [8]. ER stress-related apoptosis lead to a reduction in β-cell proliferation and regeneration, ultimately resulting in reduced insulin secretion and increased T2DM morbidity [104]. Therefore, maintaining transcriptional stability and reducing protein homeostasis loss during aging was crucial to recovering pancreatic function. It has been reported that Uro A suppresses glucolipotoxicity-induced ER stress in pancreatic beta cells [88]. However, more studies are needed on Uro A’s upstream and downstream pathways in the pancreas to improve ER stress.

### 4.4. Inhibits the Occurrence and Development of Pancreatic Tumors

High intakes of berries rich in ETs, including strawberries, pomegranates, and blueberries, were inversely associated with PDAC incidence [105]. EA, an intestinal metabolite of ellagic tannins, inhibited multiple carcinogenic pathways activated in PDAC, such as COX-2, NF-κB, and Wnt signaling, so that EA successfully arrested cell cycles and reversed epithelial to mesenchymal transition in PDAC [106]. As a downstream compound of EA, Uro A showed more potent antioxidant and anti-inflammatory properties, improving bioavailability and anti-tumor effect [107]. It has been demonstrated that the S473 phosphorylation site of AKT is activated by PI3K [108]. Uro A treatment resulted in a dose-dependent reduction in phospho-AKT (p-AKT) expression in PDAC cell lines, leading to a significant down-regulation of phospho-p70 S6 kinase (p-PS6K) expression regulated by the mTORC1 complex. Therefore, Uro A inhibited the proliferation and migration of PDAC cells and enhanced apoptosis by down-regulating the PI3K/AKT/mTOR pathway [109] (Figure 2). Furthermore, Uro A treatment also down-regulated PDK1 (the upstream target of AKT) and p-GSK3β and p-4E-BP1 (the downstream targets of AKT), suggesting that Uro A effectively inhibited the PDK1/AKT/mTOR [110]. Uro A treatment also reduced immunosuppressive tumor-associated macrophages (TAMs) and regulatory T cells in the engineered PKT mouse model of PDAC. It meant that Uro A treatment attenuated tumor growth and prolonged survival in mice by inducing changes in the immunosuppressive microenvironment of PDAC [91]. Srinivasan et al. also pointed out that Uro A inhibited AKT, PS6K, and STAT3 signaling, thereby reducing the Ki67-positive tumor cells and increasing cleaved caspase-3 expression in the pancreatic tissues of PDAC mice [92]. These results suggest that Uro A is a novel inhibitor/regulator for multi-signal pathways in PDAC and has potential in the prevention and treatment of pancreatic cancer (Table 1).

### 4.5. Protects Pancreatic β Cells

There have been many studies on the ameliorative effect of Uro A on DM and its complications. Specifically, Savi et al. first showed that Uro A recovered cardiomyocyte contractility and calcium dynamics in diabetic cardiomyopathy (DCM) rats [111]. Albasher et al. further demonstrated that Uro A prevents streptozotocin (STZ)-induced DCM in rats by activating SIRT1 expression and deacetylase activity [112]. Xiao et al. suggested that Uro A can attenuate DM-related cognitive impairment by ameliorating systemic inflammation and intestinal barrier dysfunction through the N-glycan biosynthesis pathway [113]. This conclusion was also supported by Lee et al. They pointed out that Uro A prevented DM-associated AD by reducing transglutaminase type 2 (TGM2)-dependent mitochondria-associated ER membrane (MAM) formation and maintaining mitochondrial calcium and ROS homeostasis [35]. Xu et al. indicated that Uro A ameliorated diabetic retinopathy by activating the Nrf2/HO-1 pathway to inhibit inflammation and oxidative stress [114]. Zhou et al. found that *Phyllanthus emblica* L. facilitated vascular function in STZ-induced hyperglycemia rats by regulating Akt/β-catenin signaling, mediated by the ETs metabolites [115].

Insulin resistance is one of the core mechanisms of DM. However, as a complex systemic metabolic disease, insulin resistance alone is not enough to cause DM. Islet dysfunction caused by the decrease in the total amount of islet β cells is also the key to the pathogenesis of DM. Studies have shown that β cells in T2DM can be divided into three main states: susceptibility, adaptation, and failure [116,117,118]. During insulin resistance, β cells compensate for the dysfunction by increasing insulin demand through insulin secretion [90]. When β cells fail to compensate for glucose homeostasis, hyperglycemia occurs. More importantly, EA from *Phyllanthus emblica* L. increased the size or number of β cells in diabetic rats. EA also directly increased glucose-stimulated insulin secretion from isolated islets, suggesting that EA acted directly on pancreatic β cells to exert anti-diabetic activity, thereby stimulating insulin secretion and reducing glucose intolerance [93]. Histopathological results showed that Uro A had protective effects on β cells, such as improving the pancreatic structure and increasing islet size and number. Ultrastructural damages in DM mice pancreas after Uro A treatment, including ER expansion, mitochondria swelling, cristae fracture, and myelin sheath formation, were also significantly improved [90]. We also discussed earlier that Uro A prevented β-cell apoptosis in T2DM model mice by activating autophagy and regulating the AKT/mTOR signal [88,90,102]. However, the specific mechanisms of Uro A improving β-cell structure and function to mitigate DM risk need to be explored further.

In summary, the metabolism and the roles of Uro A in ameliorating pancreatic diseases have been extensively discussed in this section (summarized in Figure 2). By clarifying Uro A’s metabolism in vivo and Uro A’s mechanisms for protecting the pancreas, it might shed new light on managing pancreatic injuries via plant-based foods rich in ETs and EA.

## 5. Conclusions

Uro A is metabolized from ETs and EA in mammals by gut bacteria and is significantly associated with systemic beneficial effects [119]. This review introduced the metabolic process of Uro A through intestinal floras and discussed the benefits of Uro A in vivo and in vitro models of health decline linked to inflammation, oxidative stress, aging, metabolic disorders, AD, and cognitive impairment. In particular, we focused on the Uro A’s mechanisms in attenuating pancreatic diseases by inhibiting inflammatory signaling pathways, activating autophagy, maintaining the mitochondrial function, and improving the immune microenvironment.

Since EA is insoluble and the gastrointestinal tract absorbs only a mere portion of it, the intestinal microbiota metabolizes most EA into urolithins, which are more easily absorbed and circulated through the bloodstream to cells and tissues [120]. However, due to the differences in age, health status, and composition of intestinal floras, the types and concentrations of produced urolithins can vary among individuals. Individuals also respond differently to exposure to urolithins or EA. Uro A has attracted attention due to its solid biological activity in the past decade. Uro A’s bioaccessibility was proven in an in vitro digestive simulation test, and toxicological studies have shown that Uro A has a good safety profile [2,121].

Moreover, there are many studies on the ameliorative effect of Uro A on DM and its complications, and most of them are mainly achieved by improving insulin resistance [112,113]. However, the effects and mechanisms of Uro A directly acting on β cells to boost glucose metabolism disorders were not discussed in detail. In addition, Uro A can also act on the pancreatic exocrine system to relieve pancreatitis or PDAC [89,92]. Several key factors support the improvement of pancreatic disease by Uro A. At first, Uro A inhibited ER stress and the TXNIP/NLRP3/IL-1β inflammation signal in β cells by modulating autophagy [88]. Uro A promoting PINK1/Parkin-mediated mitophagy to maintain mitochondrial function was also reported in pancreatic cells of diabetic mice [90]. Meanwhile, Uro A attenuates tumor growth and prolonged survival in mice by inducing changes in the immunosuppressive microenvironment of PDAC [91]. 

Uro A has beneficial effects in many tissues and is closely linked to gut microbes [122]. Intestinal microbial composition regulates the ability of ETs and EA to produce Uro A [15]. Future studies should clarify the bacterial species responsible for the urolithins conversion and investigate the relationship between Uro A, gut bacteria, and pancreatic diseases. This may shed light on the protective effects of Uro A on the pancreas, especially the exocrine pancreas. Meanwhile, considering the safety and wide sources of Uro A prepared by biological fermentation, we are supposed to look for strains that can efficiently transform ETs and EA into high-purity Uro A. We advocate for the nutritional supplementation of Uro A as an innovative way to ameliorate the function of the damaged pancreas. However, whether Uro A can delay pancreatic aging or enhance normal pancreatic function in healthy people and its mechanism remain unclear. Therefore, further studies are required to exploit the roles and mechanisms of using Uro A to protect the pancreas.

## Figures and Tables

**Table 1:** Potential effects and mechanisms of urolithin A and its precursor compounds EA on pancreatic diseases.

Disease Model	Treatment	Metabolic Response	Ref.
Spontaneous CP in male Wistar Bonn/Kobori rats	100 mg/kg BW/day orally administered with EA for 10 weeks	Attenuated pancreatic inflammation and fibrosis Increased pancreatic weight Decreased MPO activity, collagen content, TGF-β1 expression; activated PSCs and ED-1-positive cells	[85]
PSCs were isolated from rat pancreas tissue to culture activated, myofibroblast-like phenotype	1–25 μg/mL EA	Inhibited PSCs’ proliferation and migration Inhibited MCP-1 production and the expression of smooth muscle actin and collagen genes Inhibited the tyrosine phosphorylation of PDGF P-receptor Inhibited the activation of Akt and MAPKs	[86]
L-arginine induced AP in rats	85 mg/kg orally administered with EA	Decreased TOS levels Increased TAC levels Decreased TNF-α, IL-1β, and IL-6 serum levels Healed inflammatory and oxidative stress	[87]
MIN6 β-cell inflammations were induced using 25 mM glucose and 0.5 mM palmitic acid	Uro A	Inhibited TXNIP/NLRP3/IL-1β inflammation signal Modulated autophagy Down-regulated the PERK and promoted AMPK phosphorylation	[88]
Alcohol-associated CP in C56BL6/J mice	Administered during the last 3 weeks of alcohol-associated CP induction	Attenuated the severity of ACP Regulated PI3K/AKT/mTOR signaling axis	[89]
DM in male C57BL/6 mice was achieved by a HFD and intraperitoneal STZ injections	50 mg/kg BW/day orally administered with Uro A for 8 weeks	Promoted PINK1/Parkin-mediated mitophagy	[90]
Human PDAC cell lines; PDAC mice were achieved by injecting PANC1 cells into the flank of 6-week-old Fox1-nu/nu mice	0–100 μM; 20 mg/kg BW/day (5 days/week) orally administered with Uro A	Inhibited the proliferation and migration of PDAC cells Enhanced apoptosis by down-regulating the PI3K/AKT/mTOR pathway Inhibited the PDK1/AKT/mTOR pathway Reduced immunosuppressive TAMs and regulatory T cells	[91]
PKT (Ptf1a^cre/+^; LSL-Kras^G12D^; Tgfbr2^fl/fl^) mice, an aggressive genetically engineered PDAC mouse model	Orally administered with Uro A for 5 weeks	Inhibited AKT, PS6K, and STAT3 signaling Reduced the Ki67-positive tumor cells Increased cleaved caspase-3 expression	[92]
Neonatal STZ-induced non-obese T2DM rats	25–100 mg/kg BW orally administered with EA	Stimulated glucose-stimulated insulin secretion from isolated islets Decreased glucose intolerance in diabetic rats	[93]
